# TGMS in Rapeseed (*Brassica napus*) Resulted in Aberrant Transcriptional Regulation, Asynchronous Microsporocyte Meiosis, Defective Tapetum, and Fused Sexine

**DOI:** 10.3389/fpls.2017.01268

**Published:** 2017-07-20

**Authors:** Xi-Qiong Liu, Zhi-Quan Liu, Cheng-Yu Yu, Jun-Gang Dong, Sheng-Wu Hu, Ai-Xia Xu

**Affiliations:** Department of Plant Science and Technology, College of Agronomy, Northwest A&F University Yangling, China

**Keywords:** *Brassica napus*, thermo-sensitive genic male sterility, tapetum, callose, exine, glucanase, *POLLENLESS3-LIKE 2*

## Abstract

The thermo-sensitive genic male sterility (TGMS) line SP2S is a spontaneous rapeseed mutation with several traits that are favorable for the production of two-line hybrids. To uncover the key cellular events and genetic regulation associated with TGMS expression, a combined study using cytological observation, transcriptome profiling, and gene expression analysis was conducted for SP2S and its near-isogenic line SP2F grown under warm conditions. Asynchronous microsporocyte meiosis and abnormal tapetal plastids and elaioplasts were demonstrated in the anther of SP2S. The tetrad microspore did not undergo mitosis before the cytoplasm degenerated. Delayed degradation of the tetrad wall, which led to tetrad microspore aggregation, resulted in postponement of sexine (outer layer of pollen exine) formation and sexine fusion in the tetrad. The nexine (foot layer of exine) was also absent. The delay of tetrad wall degradation and abnormality of the exine structure suggested that the defective tapetum lost important functions. Based on transcriptomic comparisons between young flower buds of SP2S and SP2F plants, a total of 465 differentially expressed transcripts (DETs) were identified, including 303 up-regulated DETs and 162 down-regulated DETs in SP2S. Several genes encoding small RNA degrading nuclease 2, small RNA 2′-O-methyltransferase, thioredoxin reductase 2, regulatory subunit A alpha isoform of serine/threonine-protein phosphatase 2A, glycine rich protein 1A, transcription factor bHLH25, leucine-rich repeat receptor kinase At3g14840 like, and fasciclin-like arabinogalactan proteins FLA19 and FLA20 were greatly depressed in SP2S. Interestingly, a *POLLENLESS3-LIKE 2* gene encoding the Arabidopsis MS5 homologous protein, which is necessary for microsporocyte meiosis, was down-regulated in SP2S. Other genes that were up-regulated in SP2S encoded glucanase A6, ethylene-responsive transcription factor 1A-like, pollen-specific SF3, stress-associated endoplasmic reticulum protein 2, WRKY transcription factors and pentatricopeptide repeat (PPR) protein At1g07590. The tapetum-development-related genes, including *BnEMS1, BnDYT1*, and *BnAMS*, were slightly up-regulated in 3-mm-long flower buds or their anthers, and their downstream genes, *BnMS1* and *BnMYB80*, which affect callose dissolution and exine formation, were greatly up-regulated in SP2S. This aberrant genetic regulation corresponded well with the cytological abnormalities. The results suggested that expression of TGMS associates with complex transcriptional regulation.

## Introduction

*Brassica napus* (AACC, 2n = 38) is a young amphiploid species formed by hybridization of its ancestors *Brassica oleracea* (CC, 2n = 18) and *Brassica rapa* (AA, 2n = 20). It is an important source of edible oil and is widely grown throughout the world. Male sterilities (MS) are of importance in rapeseed because they can facilitate the production of hybrid seeds and utilization of inter-varietal heterosis on a large scale. MS exist widely in plants because pollen development can be ceased at various stages, including anther cell division and differentiation, microsporocyte meiosis, tetrad microspore release, microspore mitosis, pollen wall development, and anther dehiscence (Ma, 2005; Aya et al., 2011; Zhu et al., 2011; Sharma and Nayyar, 2016). Pollen development relies on the functions of numerous genes from both the microspore itself and sporophytic anther tissues. For example, several genes are crucial for *Arabidopsis* tapetum development and function, including *SPOROCYTELESS* (*SPL*), *EXCESS MICROSPOROCYTES 1* (*EMS1*), *GLUTAREDOXINS2* (*ROXY2*), *TAPETAL DETERMINANT 1* (*TPD1*), *DYSFUNCTIONAL TAPETUM* (*DYT1*), *TAPETUM DEVELOPMENT AND FUNCTION1* (*TDF1*), *ABORTED MICROSPORES* (*AMS*), *MALE STERILITY1* (*MS1*), and *MYB80* (*MYB103*) (Ma, 2005; Aya et al., 2011; Zhu et al., 2011; Sharma and Nayyar, 2016). In the last decade, transcriptomic studies have identified thousands of genes that are expressed in various male sterilities in cruciferous plants, including *B. oleracea* (Kim et al., 2014; Ma et al., 2015), *B. rapa* (Dong et al., 2013), *B. juncea* (Paritosh et al., 2014), and *B. napus* (Yan et al., 2013; Qu et al., 2015). Recently developed high-throughput RNA-Seq techniques are a powerful and cost-efficient tool for transcriptome profiling (Wang et al., 2012; Yan et al., 2013; Pan et al., 2014; Paritosh et al., 2014; Qu et al., 2015). These highly sensitive technologies allow accurate detection of gene expression level (Ozsolak and Milos, 2011), and RNA-Seq data show a high level of reproducibility in both technical and biological replicates (Marioni et al., 2008). Digital gene-expression tag profiling (DGE), driven by Illumina Solexa technology, is a simple and cost-efficient method to analyse transcriptome data. DGE is able to identify, quantify, and annotate expressed genes at the global transcriptome level (Tao et al., 2012; Wei et al., 2013), opening doors to higher-confidence target discovery and pathway studies.

The rapeseed thermo-sensitive genic male sterility mutant (TGMS) SP2S is a spontaneous mutant that was found in 2007 (Yu et al., 2015). It can be used as a promising pollination control system for hybrid production because it has a low critical daily temperature below 18°C, which is required for fertility transition, and male sterility can be restored by any other cultivar (Yu et al., 2015). However, genetic manipulation of TGMS was hard to perform, since TGMS SP2S is a complex trait controlled by the interaction between at least two recessive gene loci and some environmental factors (Yu et al., 2015). When a fertility-segregating population of SP2S crossed with another cultivar was employed for gene mapping, the main problem was phenotypic instability because some heterozygous plants also showed partial male sterility, similar to TGMS. Second, when the temperature changed, the fertility transition time of each individual MS plant in the segregating population was different, possibly due to the involvement of minor effect genes. Third, the chromosome collinearity between the A and C genomes and frequent crossover among homologous chromosomes (Chalhoub et al., 2014) in this allotetraploid species make gene mapping more difficult than in diploid species. It is typical of polyploid species, such as *B. napus*, that allelic variation of molecular markers can be problematic for analyses because they can be difficult to differentiate from much more abundant inter-homolog polymorphisms, which do not represent allelic variation (Bancroft et al., 2011). In our previous study, we observed anther development in SP2S under a light microscope and found that the earliest abnormality was extremely an enlarged fat tapetum formed at the stage of microsporocyte meiosis, followed by tetrad microspore abortion (Yu et al., 2015). However, due to the limited dissolution of light microscopy, detailed subcellular characterizations are still not available. In this study, therefore, we compared the anther ultrastructure between TGMS SP2S and wild-type SP2F and found abnormalities in meiotic behavior and organelle degradation. We also performed DGE to profile the transcriptome with the aim of screening important differentially expressed transcripts (DETs). We found that aberrant genetic regulation in SP2S corresponded well to abnormal cytological changes, including asynchronous meiosis, abnormal callose accumulation, delayed degradation of tetrad wall, and nexine absence. The results provided new insights into microsporogenesis in response to mild heat stress during reproductive growth.

## Methods

### Plant materials

Two NILs (near-isogenic lines) named SP2S (TGMS mutation) and SP2F (the wild-type) developed in our lab were used in this study (Figure S1). SP2S was developed after several consecutive generations of selfing from a spontaneous mutation found in the doubled haploid line SP2 in 2007. SP2F is the sibling of SP2S derived from the same genetic background (Yu et al., 2015). Seeds of the SP2S and SP2F lines were sown in a field in September, and the resulting seedlings had ~10 leaves per plant in early December when they passed vernalization under field conditions. The seedlings were transplanted to a greenhouse. To ensure complete TGMS expression, the daily average temperature in the greenhouse was maintained over 22°C and the photoperiod was longer than 14 h. We preferred to not compare the transcriptome of SP2S treated at high/low temperature because we were concerned that the plant growth rhythm would become asynchronous under two growth conditions.

### Microscopic observation

Flower buds of SP2S and SP2F plants grown in the greenhouse were collected and divided into groups according to their size. The exact developmental stage for each anther was determined by microscopic observation of their cytological characterization. Anther transverse sections or microspores samples were prepared, stained, and observed using our previous methods (Yu et al., 2015, 2016).

### DGE sequencing

Two groups of young flower buds (from microsporocyte to uni-nucleate microspore stage, bud length ≤3 mm) were collected from SP2S and SP2F plants grown in the greenhouse. They were designated as F1, F2, and F3 and S1, S2, and S3 to indicate different biological replicates. The samples were added to RNAlater solution (Ambion, TX, USA) for 24 h and stored at −80°C until further processing. Total RNAs were extracted using Trizol reagent (Invitrogen, CA, USA) according to the manufacturer's instructions and purified using a TRK1001 Kit (LC Science, Houston, TX). The quantity and purity of the total RNAs were analyzed on an Agilent Bioanalyser 2100 with an RNA 6000 Nano LabChip Kit (Agilent, CA, USA). Two groups of cDNA libraries corresponding to SP2S and SP2F, each with three biological replicates, were constructed using the Illumina Digital Gene Expression Tag Profiling Kit according to the manufacturer's protocol (Directional mRNA-Seq Sample-Preparation Part#15018460 Rev. A, October 2010). In brief, ~10 μg of total RNA with a RNA integrity number (RIN) >7.5 was subjected to Poly (A) mRNA isolation with poly-T attached magnetic beads (Thermo Fisher Scientific, CA, USA). Following purification, the mRNA was fragmented into small pieces using divalent cations under elevated temperature. The fragmented RNAs were dephosphorylated at the 3′ end by phosphatase and phosphorylated at the 5′ end by T4 polynucleotide kinase. The samples were cleaned with the RNeasy MinElute Kit (Qiagen, Hilden, Germany) following the instructions of the manufacturer. The purified RNAs were ligated with a pre-adenylated 3′ adapter, which enabled subsequent ligation of the 5′ adapter. Based on the adapter sequence, reverse transcription was followed by PCR to create cDNA constructs. The average insert size for the paired-end cDNA libraries was 300 bp (±50 bp). We performed single end sequencing on an Illumina Hiseq 2000 following the manufacturer's recommended protocol. The raw data containing adaptor sequences, tags with low quality sequences and unknown nucleotides N were filtered to obtain clean reads that were 36 nt in length.

All clean tags were aligned to *B. napus, B. rapa*, and *B. oleracea* genome sequences retrieved from NCBI (National Center for Biotechnology Information) using Bowtie2 v2.1.0 (http://sourceforge.net/projects/bowtie-bio/files/bowtie2/2.1.0/), with only a 1-bp mismatch allowed. Because many gaps and missing fragments were present in the genome assembly, we aligned the DGE tags to our previous transcriptome assembly dataset (file Trinity_rapeseed under TSA accession GDFQ00000000) containing 135,702 transcripts (unigenes) with NCBI blast hits and functional annotations. The number of perfect clean reads corresponding to each unigene was calculated and normalized to the number of Reads per Kilobase of exon model per Million mapped reads (RPKM). The low-frequency transcripts with the 3rd quartile of the total counts in all samples <10 were removed. Based on the expression levels, significant DETs among the two groups of samples were identified using a false discovery rate (*FDR*) ≤ 0.05 (DEseq package. http://www-huber.embl.de/users/anders/DESeq/) and fold-change ≥2. Hierarchical clustering of DETs was performed using Cluster 3.0 (http://bonsai.hgc.jp/~mdehoon/software/cluster/software.htm). Analysis of GO (Gene Ontology) was conducted using Gene2GO (ftp://ftp.ncbi.nih.gov/gene/DATA/gene2go.gz), and a BLAST search in the NCBI nucleotide collection database was conducted for functional classification of DETs. GO enrichment analysis was performed using a R programming script and hypergeometric test (one-tailed Fisher's exact test) to identify significantly enriched GO terms or pathways in DEGs compared to the whole genome background (Yan et al., 2013). Furthermore, the networks of down-regulated and up-regulated genes were drawn using the BiNGO plugin (Maere et al., 2005) of Cytoscape software (http://www.cytoscape.org/) using *Arabidopsis* data. The known and predicted interactions among the DETs were revealed by STRING (https://string-db.org/) based on *Arabidopsis* knowledge.

### Gene expression analysis by quantitative real-time PCR (QRT-PCR)

Some flower tissues from the SP2S and SP2F plants grown under warm conditions, including small buds that were ~1-mm length (microsporocyte stage), middle buds that were ~3 mm (uni-nucleate microspore stage), large buds that were ~5 mm (pollen mitosis stage), and anthers of middle buds and large buds that were collected simultaneously from at least five plants, with three biological repeats for each sample. RNAs were extracted, and cDNA synthesis was performed with 250 ng of total RNA using a PrimeScript RT reagent Kit (Takara, Otsu, Japan). Gene-specific primers (Table 1) were designed for the selected DETs according to the unigene sequences using the Beacon Designer 7.0 (Bio-Rad, CA, USA). Primers designed for nine genes necessary to tapetum differentiation and function (Zhou et al., 2012) were also included. Real-time PCR assays in triplicate were performed using SYBR Green PCR Master Mix (Applied Biosystems, CA, USA) on a QuantStudio 3 thermal cycler (Thermo Fisher Scientific, CA, USA). The *B. napus beta-actin7* gene was used as the internal control for data normalization, and SP2F was used as the negative control. The quantitative variation of each gene in various tissue samples of SP2S was calculated using a well-known relative quantification method 2^−ΔΔCT^ (Livak and Schmittgen, 2001). ΔΔCT = (Ct_gene_ − CT_actin_)_SP2S_ − (CT_gene_ − CT_actin_)_SP2F_. The expression level differences were estimated by variance analysis.

**Table 1:** Primers for key genes related to tapetum function and development and differentially expressed transcripts.

**Gene name**	**Forward sequence**	**Reverse sequence**	**Length (bp)**
*BnSPL*	ACTTCAACGAGGCGACAAATCTTAC	CGGGAAAAATTCGTACTCCTTCA	101
*BnROXY2*	GAGCTCGACCTCCACCCTCAT	GAAGTGCCCCCGGAGAAGT	100
*BnEMS1*	CTTGGTTGGTTGGGTTGTTCAGA	ACGTAGCATGGCCTGTTTGAAA	100
*BnTPD1*	AGGCAGCGACCGAACCTATG	TGACGTGGATCCTCGAGATGTT	99
*BnDYT1*	TGTGCCTGTTGGGATTTGAGA	AGTCACCATCACATAGTCCCTGAAGT	108
*BnTDF1*	GTGAAGAACCACTGGAACACGAA	TGAATTCCGTAAGGACCTGAGAAAC	100
*BnAMS*	CGAGATGGTGCCAGCTGAAC	GGATTGAACCAGTTGCCATCTG	101
*BnMYB80*	CCACTACACTCAATCCTCCTCAAGTC	TCAACACGTTTCTTGGTGAGCAA	100
*BnMS1*	ATGCCTCCACAAGAATGC	TCCCATCTAACACCAATCC	190
*glucan endo-1,3-beta-glucosidase A6*	TAACCCTCTGCCTAAACC	GCTCGTAACATTCTCTGC	166
*Pollenless 3-like 2*	TGCCATACAGAGGAGGAAGAC	AGAGCGTGAAGCCCAAGG	103
*bHLH25-like*	GAGGAGCAAGCCAGTCAGAG	TTCAGCCATAGTGATCTCAACAAC	189
*Thioredoxin reductase 2-like*	GGAAGCAATCAGAGCGGTTC	TCGGCGAGCACAGTTCTC	112
*Small RNA degrading nuclease 2-like*	GAAGTAAGGAACTGGATCAAGG	GGAGCACATAATAGCCACAAG	189
*Phosphoinositide phospholipase C1*	CTCTCCGTTGCCTCCTTCC	GGTGCTGCTTCTACTGTTGAG	124
*Leucine-rich repeat receptor-like serine/threonine-protein kinase*	GAGTTCCAGTAAGCAGAATCAAG	CGATGACAGAGCAGCCTATC	112
*ABC transporter E1*	CCAAGTGAAGAAGGTGTAGAGTC	AACTGAGGATGCGTGTATGC	140
*Fasciclin-like arabinogalactan protein 20*	CTTCGTTCTCTTCGCACTC	TCTTACACGGAGGACTGAG	87
*Small RNA 2′-O-methyltransferase*	GCCTTCTCCATTCGTTCTTC	GAATCCACATCTTGAATCAGTTG	198
*Serine/threonine-protein phosphatase 2A*	GTACGCCTCAACATCATAAGC	CGATTATAGCAAGACGAACTCTC	87
*Glycine-rich RNA-binding protein 1A*	TGTAACGGACCTTGGAATC	CTCTTCACTGTAAACCTACTAC	178
*Glucan endo-1,3-beta-glucosidase, acidic isoform-like*	TTGGCTTGTAACTGCTCATCTG	CCTTCTTGTTCGTCCTATCTCTTG	109
*Pollen-specific protein SF3*	TGAATCGCCGTTCTCTTCC	GTCCTCCAATCCGCCAAC	105
*Ethylene-responsive transcription factor 1A-like*	GCGTCGTTGAGGATACCGTAG	TTGTACCCTTGCTTCACTGAGAG	92
*Pentatricopeptide repeat-containing protein At1g07590*	GAGAAGAGAAGCAATGGCAGGTTC	GTTGTCGCACTCCGTGTTGTAG	103
*Probable transcription factor WRKY 70*	AGGCACCAACTCGTTCAAG	TGACAAGAGAGGAGGAGGAG	178
*BnActin7*	CGCGCCTAGCAGCATGAA	GTTGGAAAGTGCTGAGAGATGCA	101

## Results

### Abnormal microspore and pollen development in SP2S

**Figure 1:**
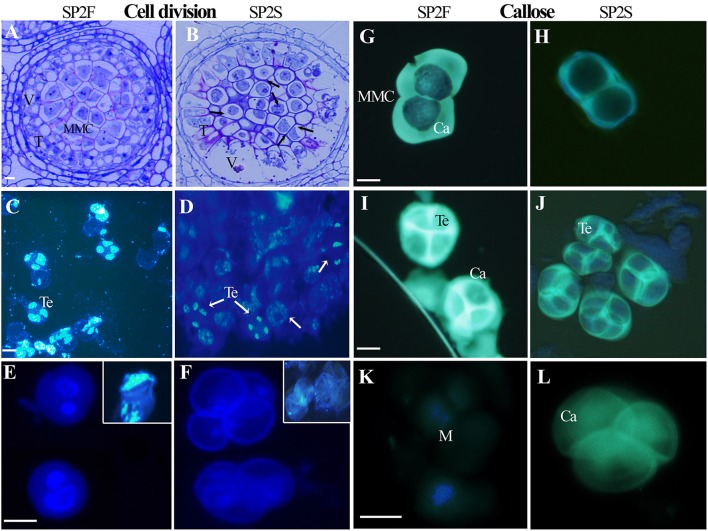
Asynchronous microsporocyte meiosis and abnormal callose layer in SP2S compared to SP2F. **(A–L)** were stained by toluidine blue, DAPI, and aniline blue, respectively. **(A)** Microsporocytes in SP2F at meiosis stage. **(B)** Different phases of meiosis exited in an anther locule of SP2S (Arrowhead showed). Fat tapetum with large vacuole was also noted. **(C)** Normal tetrads formed synchronously in SP2F. **(D)** Different phases of meiosis coexisted in one SP2S anther locule. **(E)** The bi-nucleate pollen grains of SP2F after first mitosis contained a lot of DNA with strong DAPI signal. The inset shows the clear DAPI signal in the nucleus when the cell wall was broken by slight squeeze. **(F)** The nuclear DNA of SP2S microspores was degraded, with very weak DAPI signal, and hence the microspores did not undergo mitosis. **(G)** Thick callose layer covering microsporocytes of SP2F. **(H)** Thin callose layer covering microsporocytes of SP2S. **(I)** Thick callose layer surrounding tetrad microspores in SP2F. **(J)** Thin callose layer around tetrad in SP2S. **(K)** Microspores that were released after callose dissolution, without fluorescence. **(L)** Callose walls persist even after microspore disappeared. Ca, callose; M, microspore; T, tapetum; Te, tetrad; V, vacuole. Each pair of figures had the same scale bars of 10 μm.

The developmental process of normal rapeseed anther has been well described (Yu et al., 2015, 2016), and anther abortion in SP2S is mainly due to the extremely vacuolated tapetum at the microsporocyte stage. Thus, in the present paper, we focused on new cytological phenomena. Abnormal microsporocyte meiosis, which was not noted in our previous study, was observed on both semi-thin sections of anthers and DAPI stained microsporocytes (Figure 1). The meiosis phases of all microsporocytes in the same locule should keep the same pace, and all of the tetrads formed simultaneously (Yu et al., 2016). However, meiosis in SP2S was asynchronous: some microsporocytes were still at metaphase but others entered telophase or became mid-uninucleate microspore (Figures 1B,D). A tetrad will produce four uni-nucleate microspores and each microspore will undergo mitosis and cytokinesis (Figure 1E) to become bi-cellular pollen. By contrast, the microspore of SP2S did not undergo mitosis because the nucleus degenerated at this stage, with very weak DAPI signal (Figure 1F). In addition, the callose layers covering the microsporocytes (Figure 1H) and tetrad microspores in SP2S (Figure 1J) were thinner than those in SP2F (Figures 1G,I). The tetrad wall (mainly callose layer) should be dissolved to release uni-nucleate microspore (Figure 1K) but the wall in SP2S persisted even after the microspore disintegrated (Figure 1L).

**Figure 2:**
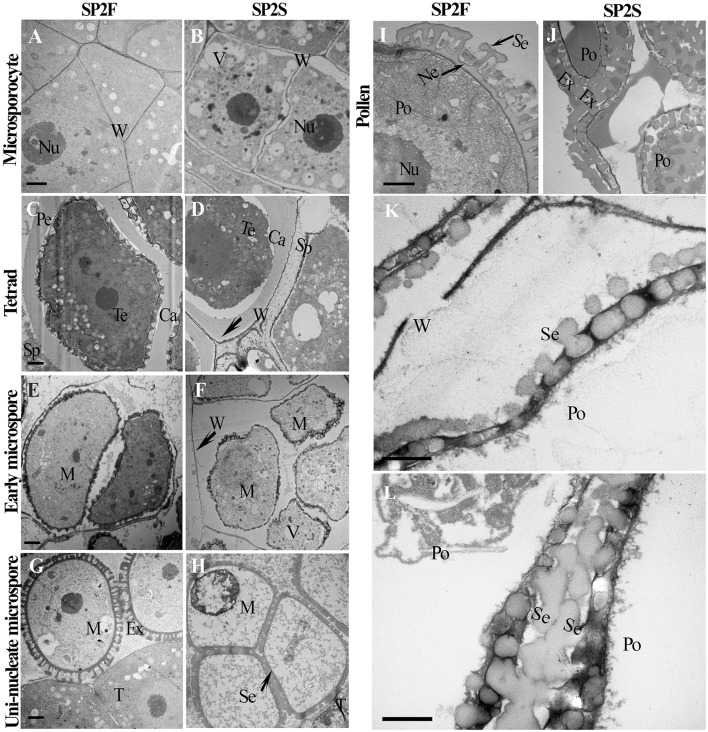
Transmission electron micrographs of microspore and pollen development of SP2F and SP2S. Developmental stage was indicated on the left side. **(A)** SP2F anther at microsporocyte stage. **(B)** Some large vacuole formed in the microsporocytes of SP2S. The primary cell wall of microsporocyte was partially degraded at tetrad stage in SP2F **(C)** but it was still maintained in SP2S **(D)**. Callose layer surrounded the tetrad in both SP2F **(C)** and SP2S, but primexine formed later in SP2S **(D)**. After callose dissolved, sporopollenin deposited on the uni-nucleate microspore surface in the SP2F **(E)** but not in SP2S **(F)** due to the barrier of residual callose and primary cell wall (arrowhead) of microsporocyte. Exine formed well on free microspore in the wild type **(G)**, but sexine fused in SP2S due to aggregation of tetrad microspores **(H)**. Pollen grain passed mitosis with well-formed exine in SP2F **(I)**; in comparison, aborted pollen had only a distorted pollen wall in SP2S **(J)**. The last two magnified images showed exine without nexine layer in SP2S **(K)** and sexine fusion of neighbor pollen grains **(L)**. Ca, callose; Ex, exine; M, microspore; Ne, nexine; Nu, nucleus; Po, pollen; Se, sexine; Sp, sporopollenin; T, tapetum; Te, tetrad; V, vacuole; W, cell wall. Each pair of figures had the same scale bars of 1 μm.

To obtain details about the ultrastructure, transmission electron microscopy (TEM) was used to compare microsporogenesis and tapetum development between the SP2S mutant and wild-type SP2F. Except for excessively vacuolated tapetal cells at the microsporocyte stage (data not shown), as shown in our previous study (Yu et al., 2015), the main differences included delayed degradation of the tetrad wall, vacuolization of uni-nucleate microspores, later deposition of exine, and absence of nexine in SP2S. The microsporocytes of both SP2F and SP2S had larger nuclei (Figures 2A,B) but primary cell wall of the latter was undulated and some large vacuoles formed in the cytoplasm (Figure 2B). After microsporocyte meiosis, the primary cell wall of microsporocyte was partially degraded in SP2F (Figure 2C) but it was still maintained in SP2S (Figure 2D). A primexine matrix (the precursor of exine) was developed on the surface of the young microspore so that the sculpturing of the microspore wall was already visible in wild-type SP2F when the microspores were still within the callose wall (Figure 2C). By contrast, primexine was undeveloped (Figure 2D) in SP2S anthers, although tapetally derived electrodense material (including sporopollenin) accumulated on the surface of the callose wall, as in *Arabidopsis ms1* mutants (Vizcay-Barrena and Wilson, 2006). In wild-type SP2F, when the microspores were freed from the tetrads after dissolution of the callose wall, each uni-nucleate microspore had a dense cytoplasm and early exine, with an initial deposition of sporopollenin (Figure 2E). In the SP2S mutant, degradation of the tetrad wall was delayed (Yu et al., 2015) and patches of electrodense material rather than distinctive baculae structure (Vizcay-Barrena and Wilson, 2006) were observed on the surface of microspores (Figure 2F). Many vacuoles formed in uni-nucleate microspores of SP2S (Figure 2F), indicating the beginning of cytoplasm degeneration. By the mid-uninucleate microspore stage, the outer layer of exine, known as sexine, formed with sporopollenin and other components (Vizcay-Barrena and Wilson, 2006) deposited on the surface of microspores in wild type (Figure 2G). By comparison, the four microspores in one tetrad were appressed in SP2S, and the cytoplasm of the microspore was degenerated; the contents of the microspore disappeared (Figure 2H). Thus, the aborted tetrad microspore should undergo no mitosis. Nevertheless, sexine formed with sporopollenin deposition at the later microspore stage, indicating that the release of sporopollenin from the tapetum to microspore still occurred in SP2S, but not at a sufficient amount or at the right time. After completion of microspore mitosis, the pollen grains contained abundant cell components and the sporopollenin polymerized (Vizcay-Barrena and Wilson, 2006), forming the characteristic exine (baculae and tectum) in the wild type (Figure 2I). In SP2S, however, the foot layer nexine did not form (Figures 2J,K) and the neighbor microspores in SP2S mutant were aggregated (Yu et al., 2015) due to exine fusion (Figure 2L). The relics of tetrad wall (callose wall and/or primary cell wall of microsporocyte) still existed around some degenerated microspores in SP2S (Figure 2J).

### Tapetal abnormality associated with TGMS

**Figure 3:**
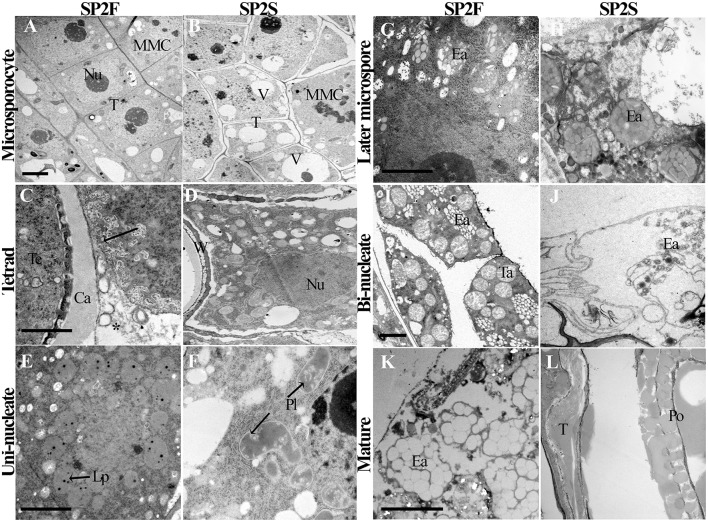
Transmission electron micrographs of tapetum development of SP2F and SP2S. At the microsporocyte stage, the tapetal cells of SP2F anther had large nucleus and thick cytoplasm **(A)** while formed many large vacuoles in SP2S tapetum **(B)**. Normal tapetum should have intensive secretory activity in wild type (**C**, Arrowhead and asterisk indicated the labyrinthine invagination and secretion granules, respectively) but inert tapetal cells in SP2S had no such labyrinthine invagination **(D)**. Wild type plastid produced oil droplets **(E)** but SP2S showed abnormal plastids with cavities **(F)**. Wild-type elaioplast contained plastoglobulus **(G)**, but the elaioplasts in SP2S quickly degenerated (**H**, Arrowhead). Wild type tapetum formed a tapetosome at a bi-nucleate pollen stage **(I)**, but the tapetum in SP2S showed no tapetosome, or some abnormal tapetosome that degraded quickly **(J)**. The tapetel organelles of SP2F degenerated when pollen grains mature **(K)** while the tapetum of SP2S withered **(L)**. Ca, callose; Ea, elaioplast; Lp, lipid droplet; MMC, microsporocyte; Nu, nucleus; Po, pollen; Pl, plastid; T, tapetum; Ta, tapetosome, Te, tetrad; V, vacuole; W, cell wall. Each pair of figures had the same scale bars of 2 μm.

Because the tapetum plays critical roles in microspore formation and pollen development, we also paid attention to the development of tapetal cells. The tapetum of SP2S showed significant abnormalities, including large vacuoles (Yu et al., 2015), persistence of the tapetal cell wall, defective plastids, and elaioplasts without lipid droplet accumulation, and absence or quick degradation of the tapetosome. The light-microscopic observation of tapetum had been showed in our previous study (Yu et al., 2015). Here we provided some details about the tapetal organelle structures that were observed under TEM. At the microsporocyte stage, the tapetal cells of SP2F anther had large nucleus and thick cytoplasm (Figure 3A) while formed many large vacuoles in SP2S tapetum (Figure 3B). At the tetrad stage, the protoplasts of tapetal cells in fertile plants contained abundant organelles (Figure 3C). The tapetal cell walls dissolved, and the small vacuoles in the tapetal cells disappeared gradually. Labyrinthine invagination appeared along the plasma membrane of the tapetal cells (Figure 3C, Arrowhead), indicating a high rate of secretion activity. By contrast, the SP2S tapetal cells showed many large vacuoles (Figure 3D). The cell walls of SP2S tapetal cells were still thick, and there was no labyrinthine invagination (Figure 3D) as in the wild type, indicating a pause in the transition of tapetal cells to secretory cells. However, excessively vacuolated tapetal cells (Yu et al., 2015) releases some contents into the locule, as shown in Figure 3D. At the uni-nucleate microspore stage, wild-type tapetal cells possessed abundant endoplasmic reticulum and secretory vesicles. Lipid droplets (Figure 3E) began to accumulate in tapetal plastids (proplast, the precursor of elaioplast) as plastoglobuli (Suzuki et al., 2013). In SP2S anthers, however, some cavities were observed in plastids (Figure 3F, Arrowhead) and the tapetal plastids and endoplasmic reticulum were significantly reduced. By the vacuolated microspore stage, tapetal cells in wild-type plants contained many elaioplasts that were full of plastoglobuli that contained nonosmiophilic bodies (Suzuki et al., 2013; Figure 3G). No obvious lipid accumulated in the SP2S tapetum; elaioplasts formed occasionally, but ruptured early (Figure 3H). At the bi-nucleate pollen stage, many tapetosomes formed (Figure 3I) in the wild-type tapetum, with abundant lipid compounds (Suzuki et al., 2013). By contrast, the tapetum in the SP2S mutant emptied, with only the relics of elaioplasts (Figure 3J). The tapetal organelles including elaioplast and tapetosome in SP2F degenerated until pollen mature stage (Figure 3K) while the taptetum of SP2S completely withered (Figure 3L).

### Profile of DGE tags

Based on the results of cytological observation, we knew the key events and critical stage of TGMS SP2S occurrence. This information was necessary for sampling at the right time in the comparative transcriptome study. To characterize the DGE profiles, we sequenced the two groups of Illumina DGE tag libraries created from the young flower buds of SP2S and SP2F plants. We obtained approximately 9 million raw reads (tags) for each library (Table S1). The raw data were deposited in the NCBI Gene Expression Omnibus (GEO) under the accession number GSE69638, in which samples F1, F2, and F3 and S1, S2, and S3 indicated the different biological replicates of two groups of samples prepared for RNA-seq. Most of the raw reads passed the quality filter, resulting in an average of 9,189,278 and 8,993,902 clean reads in SP2F and SP2S, respectively. The reads were mapped to a previous transcriptome shotgun assembly (TSA) (file Trinity_rapeseed under accession GDFQ00000000) and successfully identified an average of 108,161 and 109,523 transcripts in SP2F and SP2S, respectively (Table S1).

### Analysis of differentially expressed transcripts

To compare the differential expression patterns among the two group libraries, we normalized the count numbers and calculated the RPKM (Reads per Kilobase of transcript per Million mapped reads) values for each transcript. At total of 465 DETs (303 up-regulated and 162 down-regulated DETs), including 40 DETs specific to SP2S and 62 DETs specific to SP2F (Dataset S1), were extracted using the criteria *FDR* ≤ 0.05 and fold change ≥2. The clustering of the DETs in the two groups of samples are shown in a heatmap (Figure S2). The expression of each DET showed good reproducibility among three replicates in each sample group. Co-expression of some DETs placed in the same clustering group was expected.

**Figure 4:**
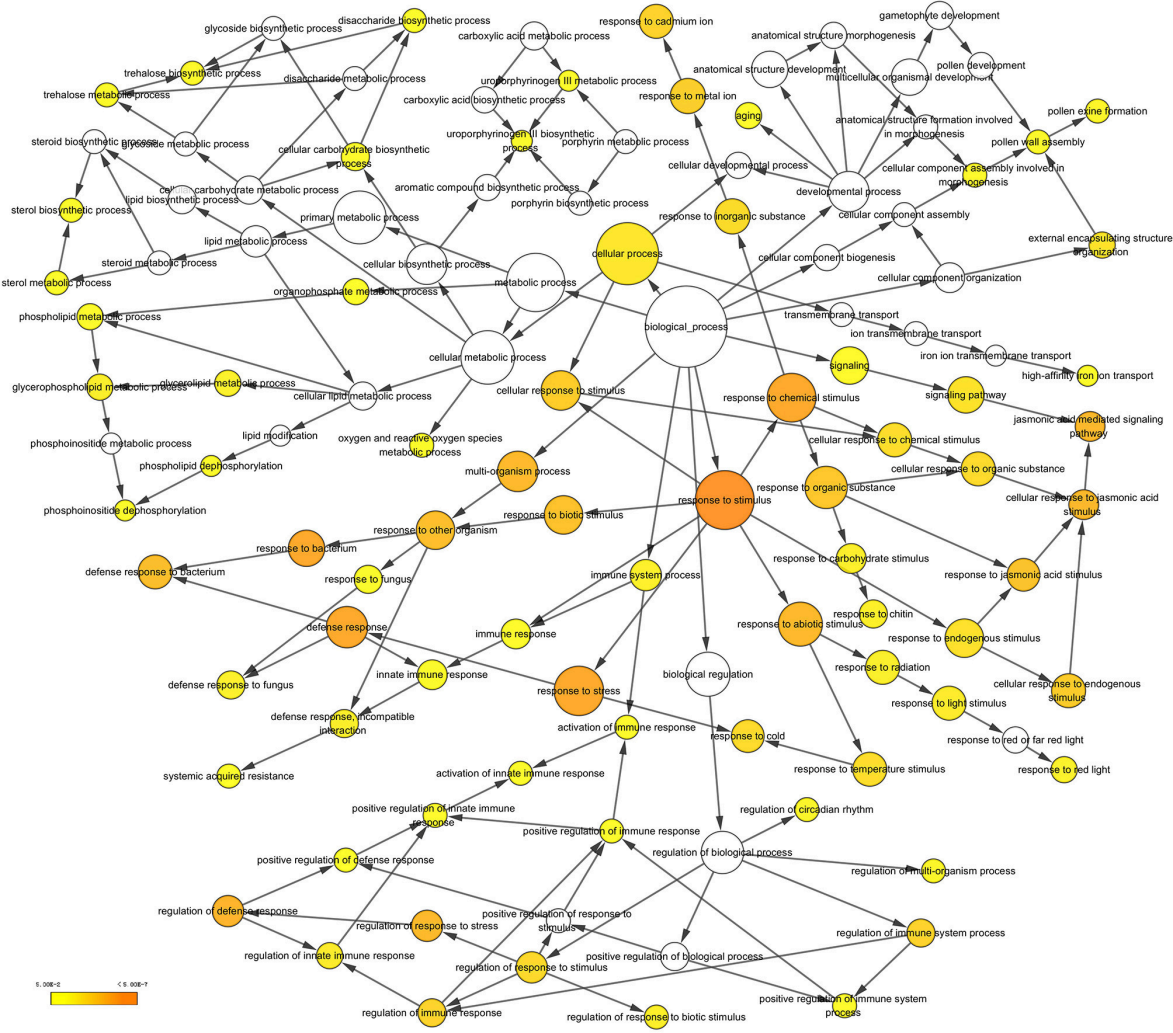
A network of up-regulated and down-regulated gene ontology (GO) terms in biological processes. The scheme was drawn using the BiNGO plugin of Cytoscape software. GOs are presented as nodes and associations are presented as edges. The significant level of node are showed by yellow color as indicated in the scale bar.

**Figure 5:**
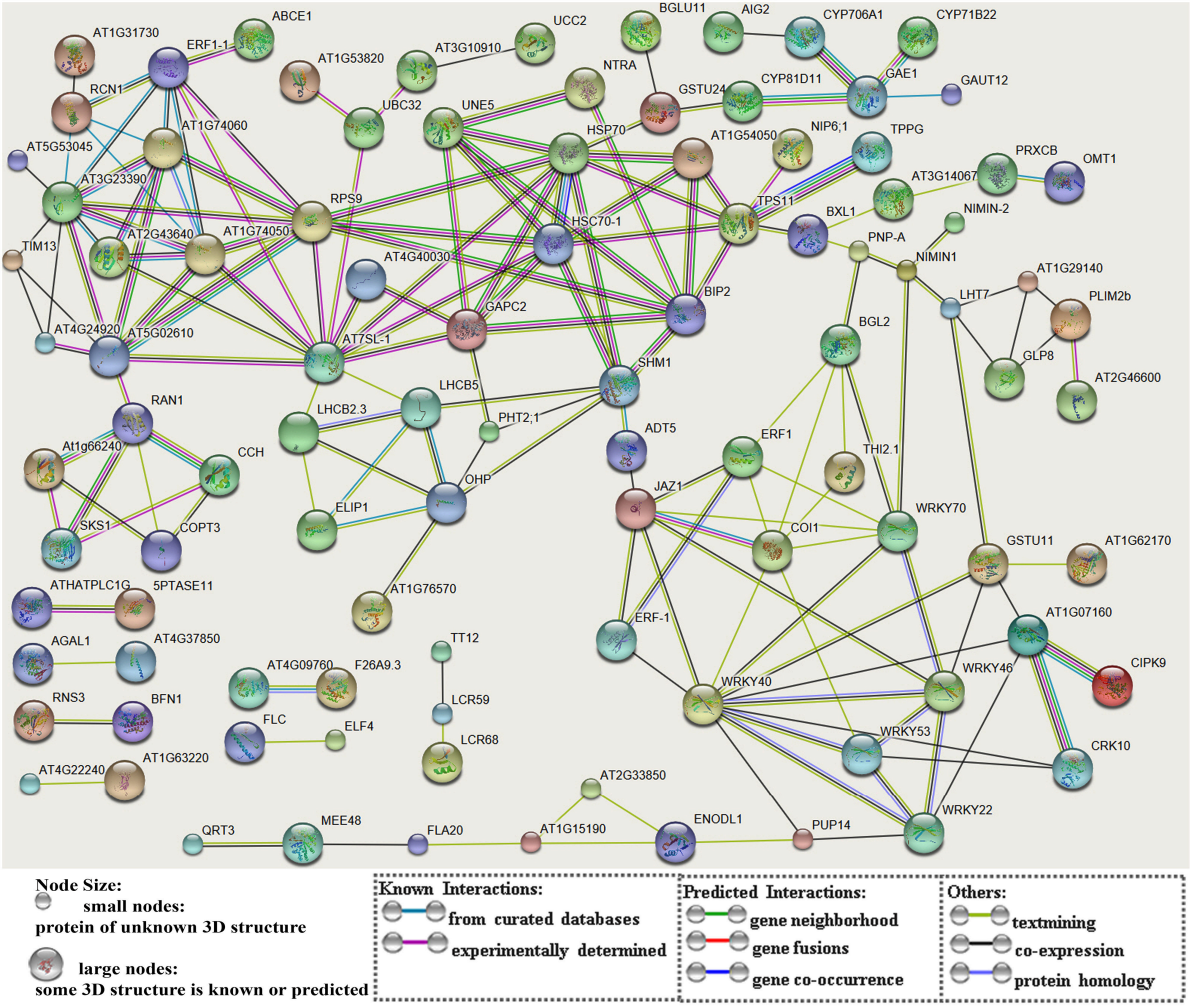
A network of protein-protein interactions based on analysis of the DETs. The scheme was drawn using the STRING software. The gene names were listed in Dataset S1. Genes are showed as nodes and interactions are presented as edges.

After searching the NCBI nucleotide database, the functions of 197 DETs were predicted with perfect BLAST matches, except for non-coding RNAs. All 50 down-regulated transcripts and the top 50 of 147 up-regulated transcripts with annotations are listed in Tables 2, 3, respectively. The DETs were matched to different GO terms (Dataset S2) using the NCBI Gene2GO database and categorized into groups based on the known or predicted functions of the corresponding GO terms. The detailed GO networks of down-regulated and up-regulated genes (Figure 4) were drawn using the BiNGO plugin of Cytoscape software. The most highly represented biological processes included defense responses to various stimulates, signaling pathways, inositol trisphosphate metabolism, thioredoxin biosynthesis, gametophyte development, pollen exine formation, and regulation of flower development, implicating intricate changes in processes of stress-response, growth, and development. We further investigated the known and predicted interactions among the DETs by STRING. The largest cluster in the derived interaction network (Figure 5) involved 85 genes and it could be divided into several groups. The group of defense response and signaling consisted of several WRKY transcription factors, SCF (COI1) E3 ubiquitin ligase complexes, TIFY10A/JAZ1, ethylene response factors ERF1 and ERF-1, and phosphatase 2C (AT1G07160). This group was linked to the group of pollen exine formation (QRT3, A6/MEE48, AT1G15190/FLA19, FLA20, E6/At2G33850, ENODL1) by purine permease PUP14. The third group was dominated by RNA polymerase II transcription subunits (HSP70, HSC70-1, and HSP70-11/BIP2), 60S ribosomal proteins (AT3G23390, AT1G74050, and AT1G74060), 30S ribosomal protein RPS9, signal recognition particle AT7SL-1, ubiquitin-conjugating enzyme UBC32, trehalose-phosphate synthase TPS11, PP2AA1/RCN1, and disulphide isomerase UNE5. Other two interesting genes UDP-glucuronate 4-epimerase GAE1 and galacturonosyltransferase GAUT12 are involved in pectin synthesis and assembly and, together with GSTU24, CYP81D11, CYP71b22, and CYP706A1, play roles in defense response. Taken together, these DETs constituted a larger network involved mainly defense responses and related regulations.

**Table 2:** Fifty annotated transcripts that are down-regulated in SP2S.

**Accession**	**Fold change**	**FDR**	**Blast hit**
comp74199_c0_seq1	−Infinite	0.0085	Pollenless 3-like 2 (loc106352295)
comp80937_c0_seq1	−Infinite	0.0119	Transcription factor bhlh25-like (LOC103860395)
comp14322_c0_seq1	−Infinite	0.0000	SLG-54 gene for S locus glycoprotein-54
comp73860_c0_seq1	−Infinite	0.0003	Ubiquitin-conjugating enzyme E2 32 (LOC103839514)
comp73769_c0_seq1	−Infinite	0.0393	Glutathione S-transferase T3-like (LOC103868979)
comp22377_c0_seq1	−Infinite	0.0000	Argonaute 9-like (LOC103851403)
comp32900_c0_seq1	−9.24	0.0000	TIFY 10A-like (LOC103872713)
comp65217_c1_seq7	−6.37	0.0000	LRR-RK serine/threonine-protein kinase At3g14840 (LOC106423522)
comp17045_c0_seq1	−6.17	0.0000	Thioredoxin reductase 2-like (LOC103874128)
comp49044_c0_seq1	−5.60	0.0000	Small RNA degrading nuclease 2 mRNA
comp73699_c0_seq1	−5.51	0.0164	AT-rich interactive domain-containing protein 3-like (LOC103845954)
comp51705_c0_seq3	−5.46	0.0022	Germin-like protein subfamily 2 member 3 (LOC103850003)
comp42223_c0_seq5	−5.20	0.0124	F-box protein At3g59000-like (LOC103830189)
comp40009_c0_seq2	−5.11	0.0000	ABC transporter E family member 1-like (LOC104765147)
comp24238_c0_seq1	−5.03	0.0000	LRR-RK serine/threonine-protein kinase At3g14840 (LOC106423522)
comp57558_c1_seq6	−4.94	0.0001	Ubiquitin-conjugating enzyme E2 32 (UBC32) (LOC103839514)
comp69590_c1_seq11	−4.59	0.0000	Vacuolar proton atpase a3-like (LOC103869355)
comp55352_c1_seq3	−4.41	0.0000	Phosphoinositide phospholipase C 1 (LOC106387427)
comp39147_c0_seq1	−4.36	0.0277	60S ribosomal protein L6-2 (LOC103831880)
comp63945_c1_seq7	−3.90	0.0000	LRR-RK serine/threonine-protein kinase At3g14840 (LOC106423522)
comp43194_c0_seq2	−3.83	0.0000	ABC transporter E family member 1-like (LOC103859527)
comp40777_c0_seq4	−3.73	0.0000	17.4 kda class III heat shock protein-like (LOC103832638)
comp60516_c2_seq17	−3.73	0.0008	Probable galacturonosyltransferase 12 (LOC103851946)
comp67754_c0_seq1	−3.34	0.0255	Copper-transporting atpase RAN1 mRNA
comp72749_c0_seq1	−3.06	0.0214	Phospholipase D beta 2 (LOC103853306)
comp58869_c0_seq14	−2.96	0.0000	NRT1/ PTR FAMILY 7.2-like (LOC103859281)
comp22809_c0_seq2	−2.96	0.0000	MADS-box protein FLC5
comp67097_c1_seq3	−2.70	0.0337	Sorting and assembly machinery component 50 homolog B
comp54689_c1_seq1	−2.61	0.0124	Histidine-rich glycoprotein-like (LOC103830387)
comp55352_c0_seq1	−2.58	0.0000	Phosphoinositide phospholipase C 1 (LOC103845299)
comp26458_c0_seq1	−2.56	0.0070	Fasciclin-like arabinogalactan protein 19 (LOC103833202)
comp65831_c1_seq1	−2.50	0.0005	Thioredoxin reductase 2-like (LOC103874128)
comp54155_c2_seq2	−2.48	0.0003	Small RNA 2′-O-methyltransferase-like (LOC103858486)
comp42529_c0_seq1	−2.44	0.0220	Putative F-box protein At4g10740 (LOC106297876)
comp65727_c0_seq14	−2.36	0.0005	Histone deacetylase HDT3-like (LOC106326328)
comp65831_c1_seq2	−2.33	0.0031	Thioredoxin reductase 2-like (LOC103874128)
comp69180_c1_seq3	−2.32	0.0295	Coronatine-insensitive protein 1-like (LOC103865755)
comp49237_c0_seq2	−2.24	0.0086	65 kda regulatory subunit A alpha isoform of phosphatase 2A
comp63945_c1_seq5	−2.04	0.0438	LRR-RK serine/threonine-protein kinase At3g14840 (LOC106349287)
comp58296_c0_seq2	−1.97	0.0002	Glycine-rich RNA-binding protein GRP1A (LOC106410966)
comp45937_c0_seq4	−1.94	0.0280	Zinc finger BED domain-containing RICESLEEPER 1 (LOC103869698)
comp57362_c0_seq8	−1.65	0.0066	EARLY FLOWERING 4-like (LOC103865763)
comp66682_c0_seq7	−1.62	0.0291	Neutral alpha-glucosidase C-like (LOC103832252)
comp57483_c0_seq1	−1.50	0.0007	Monocopper oxidase-like protein SKS1 (LOC103852980)
comp61706_c0_seq3	−1.49	0.0385	Multiple organellar RNA editing factor 1, mitochondrial
comp48895_c1_seq1	−1.36	0.0385	Putative fasciclin-like arabinogalactan protein 20 (LOC103851072)
comp49466_c0_seq2	−1.29	0.0444	Probable alpha,alpha-trehalose-phosphate synthase 11 (LOC103837918)
comp65401_c0_seq1	−1.28	0.0163	LTV1 homolog (LOC103860640)
comp69719_c0_seq3	−1.25	0.0371	Glycosyltransferase (At4g01210) mRNA
comp65977_c0_seq8	−1.20	0.0412	Phosphoenolpyruvate carboxylase (PEPC) 4-like (LOC103840598)

**Table 3:** Top 50 annotated transcripts that are up-regulated in SP2S.

**Accession**	**Fold change**	**FDR**	**Blast hit**
comp85455_c0_seq1	Infinite	0.0000	60S ribosomal protein L6-3-like (LOC104751278)
comp39723_c0_seq1	Infinite	0.0237	RING-H2 finger protein ATL60 (LOC103848508)
comp38154_c0_seq1	Infinite	0.0004	Defensin-like protein 182 (LOC103829127)
comp28797_c0_seq1	Infinite	0.0001	Putative F-box protein At1g70960 (LOC103854855)
comp36113_c0_seq1	9.30	0.0000	Nardilysin-like (LOC103854597)
comp47748_c0_seq2	6.56	0.0001	Probable mediator of RNA polymerase II transcription subunit 37e
comp452_c0_seq1	6.53	0.0001	Glucan endo-1,3-beta-D-glucosidase-like (LOC103873744)
comp78010_c0_seq1	6.05	0.0078	*Raphanus sativus* D81Rfo (restorer-of-fertility)
comp40628_c0_seq2	5.76	0.0001	Early nodulin-like protein 1 (LOC103864510)
comp59887_c1_seq1	5.40	0.0140	Beta-glucosidase 11 (LOC103844557)
comp84808_c0_seq1	5.37	0.0174	Extensin-3-like (LOC103830588)
comp67571_c0_seq4	5.32	0.0000	NSF attachment protein (LOC103858222)
comp48766_c0_seq6	5.31	0.0191	Putative BTB/POZ domain-containing protein DOT3 (LOC103846825)
comp78577_c0_seq1	5.25	0.0091	Myosin IB heavy chain (LOC103852039)
comp55196_c0_seq1	4.64	0.0000	Lysine histidine transporter-like 7 (LOC103831015)
comp45715_c0_seq1	4.51	0.0002	Trichome birefringence-like 40 (LOC103857356)
comp47956_c0_seq1	4.43	0.0075	Ribonuclease 3 (LOC103829206)
comp63008_c0_seq4	4.26	0.0047	Glutathione S-transferase U24 (LOC103872504)
comp79895_c0_seq1	4.22	0.0244	TRANSPARENT TESTA 12-like (LOC103873970)
comp69320_c0_seq6	3.79	0.0000	Subtilisin-like protease (LOC103870071)
comp43787_c0_seq1	3.68	0.0204	HVA22-like protein e (LOC103857274)
comp56538_c0_seq1	3.50	0.0061	Endonuclease 1-like (LOC103843249)
comp52020_c1_seq1	3.14	0.0000	Glucan endo-1,3-beta-glucosidase A6 (LOC106449427)
comp66720_c0_seq13	3.07	0.0342	RING-H2 finger protein ATL72 (LOC103832912)
comp52222_c0_seq7	2.90	0.0014	Pollen-specific protein SF3 (LOC103843810)
comp59212_c2_seq4	2.90	0.0000	Ethylene-responsive transcription factor 1A-like (LOC103834016)
comp61627_c1_seq6	2.88	0.0140	N-acetyltransferase ycf52 (LOC103857845)
comp45701_c1_seq2	2.83	0.0002	Beta-D-xylosidase 1-like (LOC103837284)
comp46536_c0_seq3	2.82	0.0405	Pollen-specific protein SF3 (LOC103843810)
comp59887_c3_seq10	2.80	0.0003	Beta-glucosidase 11 (LOC103844557)
comp56300_c0_seq7	2.78	0.0000	Ethylene-responsive transcription factor 1A-like (LOC103855430)
comp44369_c0_seq1	2.72	0.0000	NAC domain-containing protein 67-like (LOC103850060)
comp56687_c1_seq2	2.68	0.0159	Probable phosphatase 2C 2 (LOC103843974)
comp58831_c0_seq2	2.68	0.0000	WRKY transcription factor 70 (WRKY70)
comp37552_c0_seq1	2.67	0.0113	Bnaa.FRI.a FRIGIDA-like protein
comp37512_c0_seq1	2.67	0.0137	Probable purine permease 14 (LOC103835832)
comp47600_c0_seq2	2.60	0.0000	EKC/KEOPS complex subunit LAGE3-like (LOC103857106)
comp50768_c0_seq2	2.59	0.0002	Copper transporter 3-like (LOC103845351)
comp59887_c3_seq22	2.56	0.0024	Beta-glucosidase 11 (LOC103844557)
comp56995_c0_seq1	2.52	0.0000	Pentatricopeptide repeat-containing PPR At1g07590(LOC103843443)
comp55264_c0_seq1	2.51	0.0486	Probable non-inhibitory serpin-Z5 (LOC103857162)
comp55050_c0_seq1	2.48	0.0299	Type I inositol 1,4,5-trisphosphate 5-phosphatase 11 (LOC103843905)
comp47848_c0_seq1	2.42	0.0001	Aquaporin NIP6-1 (LOC103832459)
comp64567_c0_seq1	2.41	0.0000	Polygalacturonase QRT3-like (LOC106365935)
comp59887_c3_seq32	2.40	0.0030	Beta-glucosidase 11 (LOC103844557)
comp56995_c0_seq5	2.32	0.0000	Pentatricopeptide repeat-containing PPR At1g07590 (LOC103843443)
comp55686_c0_seq2	2.30	0.0001	Cannabidiolic acid synthase-like 1 (LOC103858400)
comp59887_c3_seq31	2.28	0..0156	Beta-glucosidase 11 (LOC103844557)
comp49874_c0_seq1	2.27	0.0150	NIM1-INTERACTING 1-like (LOC103843751)
comp59761_c1_seq8	2.25	0.0043	NAC transcription factor 29-like (LOC103845998)

**Figure 6:**
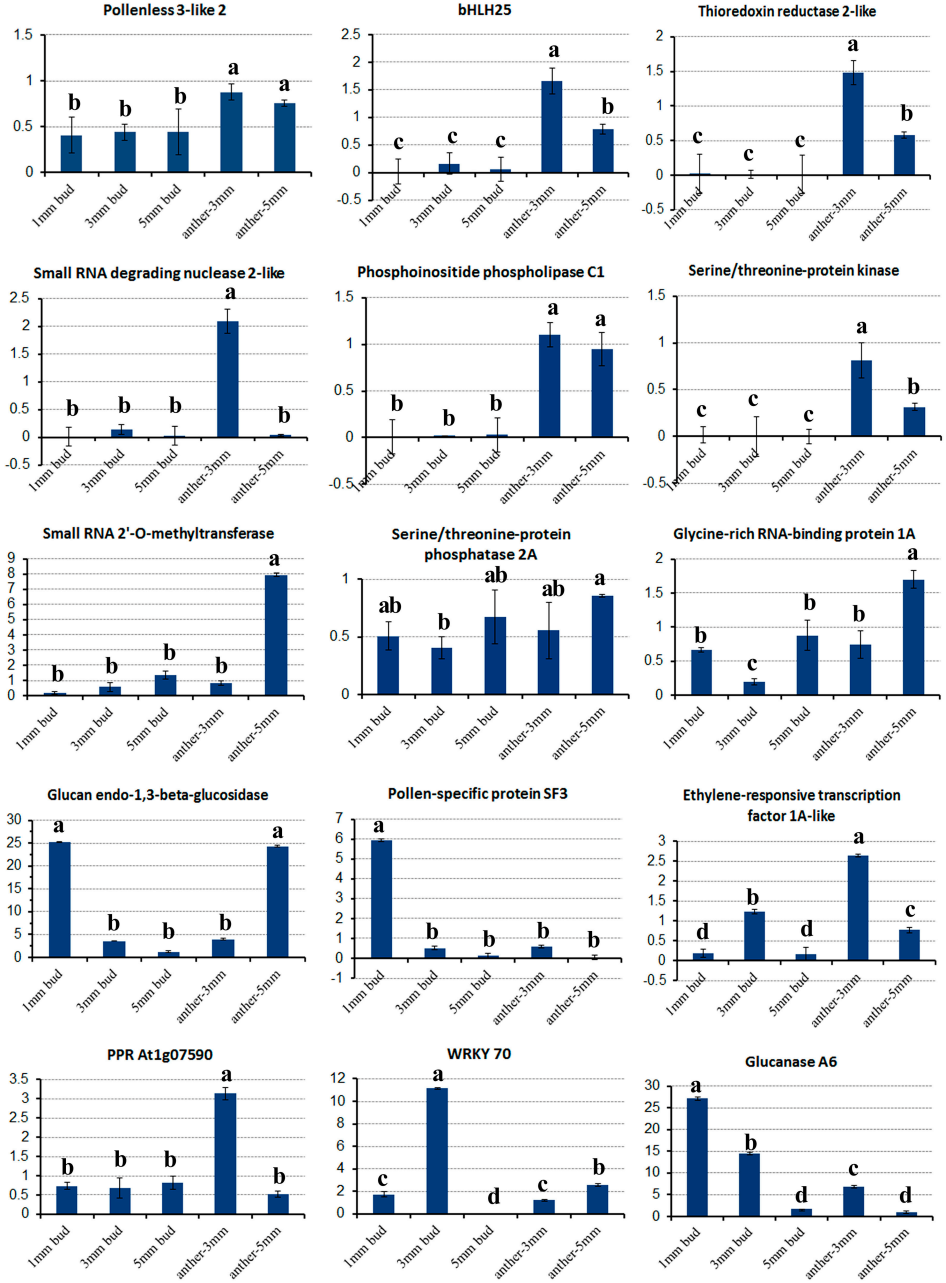
Relative expression of key differentially expressed transcripts in different tissues of SP2S compared to SP2F. Values were estimated from 2^−ΔΔCT^ values in qRT-PCR. The expression differences among different tissues at a confidence level of 95% were indicated by letters (i.e., a, b, c) and the standard deviation for each sample with three replicates was indicated by error bars.

**Figure 7:**
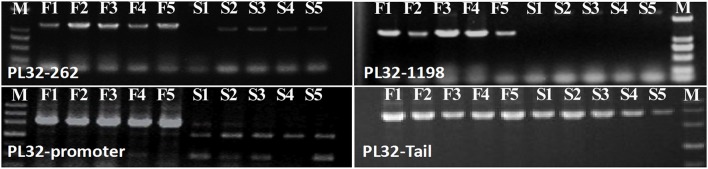
Profile of PCR amplified in SP2F (F1–F5) and SP2S (S1–S5) of four pairs of primers designed for *Pollenless3-like 2*. M, DNA marker.

In the gene lists in Tables 2, 3, we found no TGMS or TGMS-regulated genes reported by other groups, except for *POLLENLESS3-LIKE 2*, largely because until now, few environment-sensitive genes and regulation networks have been identified. In the lists, however, there should be some genes that were co-expressed with the TGMS gene or regulated by TGMS directly/indirectly. Since TGMS SP2S is a recessive mutant, down-regulated/loss-of-function genes are of greater interest than up-regulated/gain-of-function genes. Interestingly, a transcript (comp74199_c0_seq1) encoding the POLLENLESS3-LIKE 2 protein, which participates in microsporocyte meiosis (Glover et al., 1998), was absent in the SP2S transcriptome. Its relative expression level in SP2S, represented by qRT-PCR, was much lower than in SP2F (Figure 6). *POLLENLESS3* is also known as *MALE STERILITY5*/*THREE DIVISION MUTANT1* (*MS5*/*TDM1*) (Bulankova et al., 2010), and the *ms5-2* mutant of *Arabidopsis* showed an environment-sensitive male sterility phenotype (Glover et al., 1998). The sequences of the PCR products in SP2F, which were amplified using several primers designed for wild-type *POLLENLESS3-LIKE 2* (Table 4), showed a highly identical CDS, but very different promoters (unpublished) than the NCBI deposited sequence (LOC106352295) of cultivar ZS11, but the counterpart of *POLLENLESS3-LIKE 2* was missing and could not be amplified in SP2S. The fore-ends of the *POLLENLESS3-LIKE 2* DNA sequence, including PL32-Promotor, PL32-1198, and PL32-262, could not be amplified in SP2S, whereas the back end PL32-Tail was found in all samples (Figure 7). Therefore, the sequence of *POLLENLESS3-LIKE 2* should contain a long mutation zone in SP2S and will be very interesting for future study.

**Table 4:** Information of the primers designed for *POLLENLESS3-LIKE2*.

**Primer name**	**Sequence of left primer**	**Sequence of right primer**	**Product size**	**Nucleotide position from ATG codon**
PL32-1198	CTCCATTGACTCTCCCCAAA	CACAACTCAACATTCCCATTGT	1198	−21 to 1,176
PL32-262	GCCATAGTGATGAAGCAGCAA	AGGCTCTTGGGTTTAGACAGG	1088	240 to 1,328
PL32-Promoter	CGGACGTTAATTTTGGGTGT	GATACCGAGAGGCTTTGCAG	1747	−1,701 to 46
PL32-Tail	CACCGGACAACAACAAGATG	ACCCACACTCCATTGCTTTC	654	742 to 1,396

Fourteen other important DETs listed in Tables 2, 3 were also chosen for gene expression analysis via real-time PCR. The results showed that, in small buds, the relative expression levels of all of the selected DETs were in line with the DGE profiling data (Figure 6), although the expression patterns of these genes changed with sample age (Figure 6). In general, expression of genes encoding the regulatory subunit alpha isoform of serine/threonine-protein phosphatase 2A (PP2AA1), bHLH25, thioredoxin reductase 2-like (NTR2), small RNA degrading nuclease 2-like (SDN2), leucine-rich repeat receptor protein kinase (LRR-RK), phosphoinositide phospholipase C1, ABC transporter E1, small RNA 2′-O-methyltransferase, GRP1A, and MADS-box FLC5 were down-regulated. Four selected DETs that were up-regulated in SP2S encode pollen-specific protein SF3, probable transcription factor WRKY70, glucanases A6, and glucan endo-1,3-beta-glucosidase-like (LOC103873744). The other two selected DETs encode ethylene-responsive transcription factor 1A-like and pentatricopeptide repeat-containing protein (PPR) At1g07590 showed increased expression in the anther of 3-mm-long buds (Figure 6). We also found that some genes well-known to encode transcription factors that are involved in stress-response and defense, including *WRKY40, WRKY46, WRKY53, WRKY DQ209287.1*, and *NAC67* were up-regulated. In addition, the transcription factor genes *MYB4, signal recognition particle* (*SRP*), and *stress-associated endoplasmic reticulum 2-like* as well as three genes for subunit 37 of the probable mediator of RNA polymerase II (heat shock protein 70 family) were also up-regulated in SP2S. Some genes maybe co-expressed with the TGMS gene or regulated by TGMS directly/indirectly.

### Expression analysis of nine key tapetum preferential genes

**Figure 8:**
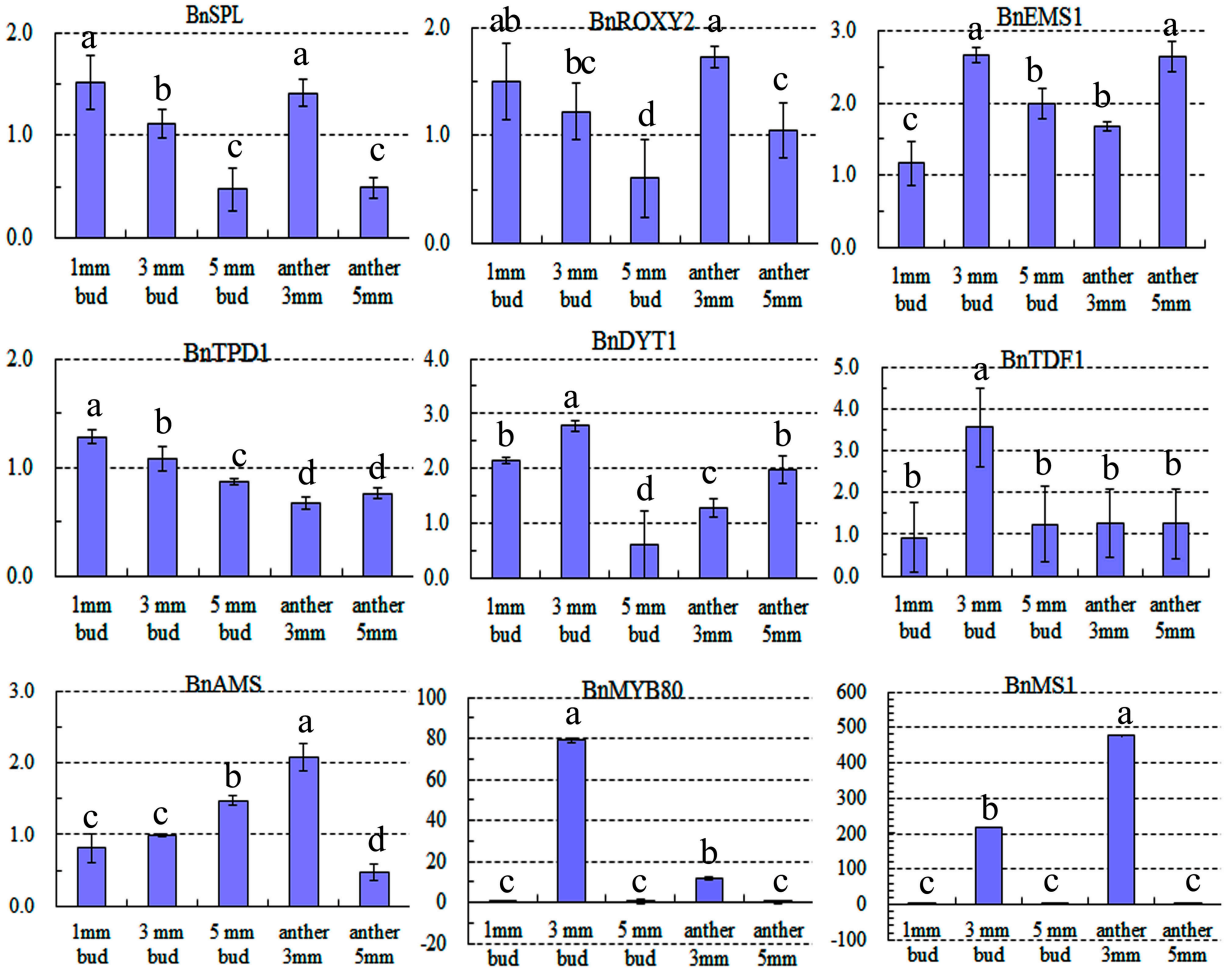
Relative expression level of nine key genes crucial for microsporocyte and tapetum development in different tissues of SP2S compared to SP2F. Values were estimated from 2^−ΔΔCt^ values in qRT-PCR. Expression differences among different tissues were indicated by letters a, b c, and d at a confidence level of 95%, and the standard deviation for each sample with three replicates was indicated by error bars. These genes were homologous to *Arabidopsis SPOROCYTELESS* (*SPL*), *EXCESS MICROSPOROCYTES 1* (*EMS1*), *CC-TYPE GLUTAREDOXINS1* (*ROXY2*), *TAPETAL DETERMINANT 1* (*TPD1*), *DYSFUNCTIONAL TAPETUM* (*DYT1*), *TAPETUM DEVELOPMENTAND FUNCTION1* (*TDF1*), *ABORTED MICROSPORES* (*AMS*), *MALE STERILITY1* (*MS1*), *and MYB80*, respectively. *MS1* and *MYB80* were highly up-regulated.

Expression of nine key genes (Zhou et al., 2012) that are crucial for tapetum development was also detected by qRT-PCR. Expression differences in the 1-mm small buds were difficult to detect (Figure 8), which may explain why there were no DETs found by DGE tag profiling. The upstream genes, including *BnSPL, BnTPD1*, and *BnROXY2*, were not affected in TGMS SP2S, except that *BnEMS1* was up-regulated (Figure 8) in middle and large buds. Three key genes, including *BnTDF1, BnAMS*, and *BnDYT1*, which control the later stage of tapetal development, were also slightly up-regulated in 3-mm-long buds or anthers. Surprisingly, expression of *BnMS1* and *BnMYB80*, whose proteins influence callose dissolution and exine formation indirectly by regulating tapetum development (Vizcay-Barrena and Wilson, 2006; Zhang et al., 2007), were greatly up-regulated in 3-mm-long floral buds (Figure 8) that correspond well to difficult dissolution of tetrad wall and delayed formation of exine.

## Discussion

### Asynchronous meiosis leads to abnormal tetrad microspores

Using cytological observation, we focused on several key events in male sterility and narrowed the window for transcriptome sampling. As a supplement to our previous microscopic observation, the electron microscopic study and microsporocyte staining helped to elucidate the detailed cellular events associated with TGMS SP2S. The phenotype of anther abortion in SP2S showed characteristics of both asynchronous meiosis of microsporocytes and “fat tapetum” (Sanders et al., 1999), different from other male sterilities reported in rapeseed (Dun et al., 2011; Xin et al., 2016). Several meiosis-specific gene sub-networks, such as the transition from mitosis to meiosis (RNA polymerase, double-stranded RNA binding, ribonucleoprotein), homologous pairing and synapsis, meiotic replication and chromosome structure control (Flap endonuclease), and meiotic progression, have been identified (Aya et al., 2011; Sharma and Nayyar, 2016). A model has been proposed in which meiotic progression in *Arabidopsis* pollen mother cells is driven by unidentified cyclins and cyclin-dependent kinase activity that is modulated by regulatory interactions between THREE DIVISION MUTANT1 (TDM1/MS5), TARDY ASYNCHRONOUS MEIOSIS (TAM/CYCA1;2), and SUPPRESSOR WITH MORPHOGENETIC EFFECTS ON GENITALIA7 (SMG7) (Bulankova et al., 2010). TDM1 is a tetratricopeptide repeat domain-containing protein that involves the regulation of cell division after male meiosis II to facilitate exit from meiosis and transition to G1 (Ross et al., 1997). The *POLLENLESS3-LIKE2* gene, which is conserved in SP2F but partially absent in SP2S, is the homolog of the *TDM1*/*MS5* gene in *Arabidopsis*. The *Arabidopsis ms5-2* mutant also showed a phenotype of environmentally sensitive male sterility, chromatin recondensation and stretching after meiosis II and was characterized by a dense microtubule network connecting haploid nuclei in the tetrad stage, which rearranged into four bipolar spindles as chromatids recondensed, as if haploid nuclei entered a third meiotic division (Glover et al., 1998). Although, we did not find a third cell division phenomenon in SP2S, there was abnormal regulation of some meiosis-specific genes, which led to asynchronous meiosis. Except for abnormal meiosis, the tetrad microspores of SP2S seemed undergo no mitosis, suggesting that TGMS might affect the completion of mitosis. This also intimated the involvement of more cell cycle related genes, for instance, down-regulation of *PP2AA1*, which encodes a 65 KD regulatory subunit alpha isoform of Serine/threonine-protein phosphatase 2A is required for proper chromosome segregation and centromeric localization in mitosis (Zhou et al., 2004).

### Abnormal tapetum affect microgamete development

In most cases, the critical male gametophytic stage that is most sensitive to abiotic stress often coincides with the meiosis to microspore transition stage (Parish et al., 2012; De Storme and Geelen, 2014). Both the anther ultrastructure and transcriptome profile showed remarkable changes in SP2S in responding to moderate heat stress at this stage. Upon exposure to heat stress, anthers typically show premature disappearance of the tapetum, together with severe alterations in microspore development, as in barley (Abiko et al., 2005), TGMS rice (Ku et al., 2003; Shi et al., 2003), and rapeseed reverse TGMS (Yu et al., 2016). During normal male gametogenesis, when the tapetum becomes secretory, it is strongly metabolically active and serves as a nutritive source by providing essential elements and energy to neighboring microspores and enzymes to release of microspores from the meiotic tetrad and cell wall components to construct the pollen exine layer, such as sporopollenin (Ariizumi and Toriyama, 2011). Once the tapetum transition is blocked, tapetal cells become enlarged and abnormally vacuolated, followed by aberrant microspore separation and abnormal pollen-wall development (Abiko et al., 2005; Ariizumi and Toriyama, 2011; Parish et al., 2012). The extreme vacuolated tapetal cells in SP2S indicated a halt or slowing of the transition to a secretory-type tapetum and attenuated or partially lost some tapetal functions that provide essential materials for microspore development.

### Later degradation of the tetrad wall leads to exine fusion

Several studies have observed clamped, tetrad-shaped spores in heat-stressed male gametogenesis in *Arabidopsis*, the cause of which was hypothesized to be persistence of tetrad wall (Kim et al., 2001; Rhee et al., 2003; De Storme and Geelen, 2014). Our present results also showed that delayed degradation of tetrad wall (callose wall and/or primary cell wall microsporocyte) led to aggregated microspores in SP2S and sexine fusion. The abnormal exine, including nexine absence and sexine fusion, reported in this study is the first case reported in *Brassica* species. As it contains callose, cellulose and pectin, the tetrad wall should be dissolved by several tapetum enzymes, including beta-1,3-glucanase and polygalacturonase (Rhee et al., 2003; Li and Wu, 2012). Our transcriptomic profiling and real-time PCR results showed that *glucanase A6, three* transcripts (comp52020_c1_seq1, comp52644_c0_seq1, and comp452_c0_seq1) encoding beta-1,3-glucosidase, and the *QRT3* gene (comp64567_c0_seq1) encoding polygalacturonase were obviously up-regulated. In addition, both microscopic and ultramicroscopic observations demonstrated abnormal callose layer surrounding tetrad microspores and even pollen (Yu et al., 2015). Similarly, the *Arabidopsis cdm1* mutant also led to incomplete degradation of callose layer and greatly up-regulated *A6* and *MYB80*, but down-regulated callose synthase gene *CalS5* and *CalS12* (Lu et al., 2014). Their results suggested a contradiction between reduced accumulation and delayed degradation of callose. Expression of *AtMYB80* and *A6* was thought to be activated precociously (Lu et al., 2014), somehow affecting the expression of *CalS5* and *CalS12* and leading to reduced callose accumulation. It seemed that up-regulation of glucanase genes may not always mean quick degradation of callose wall. It should be noted that the dynamic balance process between callose accumulation and dissolution was affected by at least three factors: the amount of callose degradation enzymes, the structure and components of tetrad wall, and the synthesis rate of cell wall materials. In fact, increased expression of *cellulose synthase A catalytic subunit, cellulose synthase D2*, and callose synthase gene *GSL2/CalS5, GSL10/CalS9*, and *GSL12/CalS3*, was found in SP2S in another study by our group (unpublished). Thus, the requirement for elevated expression of the five genes encoding callose wall degradation enzymes indicated difficulty or delay in tetrad wall degradation. The delay of tetrad wall degradation in SP2S maybe resulted from an imbalance between synthesis and degradation of the callose, cellulose, or pectin.

The pollen exine pattern appeared to be dependent on at least three major developmental processes: primexine formation, callose wall formation, and sporopollenin deposition (Ariizumi and Toriyama, 2011). Sporopollenin is synthesized via catalytic enzyme reactions in the tapetum, and both the primexine and callose walls provide an efficient substructure for sporopollenin deposition (Ariizumi and Toriyama, 2011). Thus, the abnormal primexine and callose walls in SP2S will also affect exine formation. *AtMYB80* (Zhang et al., 2007) and *MS1* (Vizcay-Barrena and Wilson, 2006) play important roles in the regulation of callose dissolution and primexine formation, respectively. Expression of *BnMYB80, BnMS1*, and *beta-1,3-glucanase A6* was abruptly up-regulated at the later microspore stage in SP2S, suggesting abnormalities of callose dissolution and sporopollenin secretion. However, the formation of sexine on the aborted microspore suggested that tapetum could synthesize some sporopollenin before it degenerated.

Moreover, secretion of such materials from abnormal tapetal cells, including lipid compounds and AGPs, also affected the formation of exine, as indicated by the lack of pollen nexine and abnormal tapetal plastids and elaioplasts in SP2S. In cruciferous plants, two specific organelles—elaioplasts and tapetosomes—play a central role in accumulating the components of the pollen coat (Suzuki et al., 2013). Sexine is predominantly composed of sporopollenin, which consists of fatty acid derivatives and phenolic compounds, while nexine is composed of arabinogalactan proteins (AGPs) (Li and Wu, 2012), which appear to be regulated by the tapetal AT-HOOK protein (Lou et al., 2014). The lack of nexine in SP2S suggested depletion of some genes for AGPs synthesis. We found down-regulation of the fasciclin-like arabinogalactan proteins FLA19 and FLA20, which are a subclass of arabinogalactan proteins (AGPs) that have, in addition to predicted AGP-like glycosylated regions, putative cell adhesion domains known as fasciclin domains (Li and Wu, 2012). Thus, the function of *FLA19* and *FLA20* in SP2S requires further study. Nevertheless, the lack of nexine may not be the main reason for microspore abortion because it occurred at a later stage of microspore degeneration.

### Other genes involved in anther abortion of SP2S

Many genes controlling stamen and pollen development have been identified, and their specific roles have been characterized (Ma, 2005; Aya et al., 2011; Zhu et al., 2011; Sharma and Nayyar, 2016). For higher plants, environment-sensitive male sterilities are difficult to decipher due to complex gene-environment interactions and phenotypic variation. Numerous studies have attempted to map the genes associated with TGMS or photoperiod-sensitive genic male sterility (PGMS), but only a few, in species with simple genomes, have been successfully cloned (Ding et al., 2012; Zhou et al., 2014; Fan et al., 2016). These studies in rice suggest that abnormal degradation and accumulation of some RNAs, for examples, *Ub*_*L*40_ mRNA, long non-coding RNA and small-interfering RNA, play important roles in the occurrence of TGMS and PGMS (Ding et al., 2012; Zhou et al., 2014; Fan et al., 2016). Our DGE profiling provided a snapshot of the complex transcriptional network that operates TGMS. However, it is difficult to anchor the trigger of the male sterility gene itself by this means, although global differential expression between the male sterile mutant and wild type is useful to unveil genes associated with anther abortion. Fortunately, the aberrant genetic regulation in SP2S corresponded well to abnormal behaviors, including asynchronous meiosis of microsporocyte, delayed dissolution of the tetrad wall, a fused sexine, and premature breakdown of the fat tapetum. This knowledge is useful to narrow the scope of interesting DETs and genes.

Some down-regulated DETs, such as bHLH25, thioredoxin reductase 2-like, SDN2, Small RNA 2′-O-methyltransferase, Phosphoinositide phospholipase C1, ABC transporter E1, LRR-RK, and GRP1A-like genes, merit further study. We determined the possible functions of these proteins based on annotations in Uniprot (http://www.uniprot.org). For example, bHLH 25 is specifically expressed in flowers and is induced by ethylene and jasmonic acid treatments. Thioredoxin reductase 2-like is a mitochondrial enzyme that catalyses the reduction of thioredoxin and is implicated in defense against oxidative stress. Small RNA-degrading nuclease 2 (SDN2) has 3′–5′ exonuclease activity that degrades single-stranded small RNAs. Small RNA 2′-O-methyltransferase protects the 3′-end of sRNAs from uridylation activity and is involved in plant development through its role in small RNA processing. The ABC transporter E1 is an ATP-binding cassette and utilizes the energy of ATP binding and hydrolysis to transport various substrates across cellular membranes. GRP1A performs RNA chaperone functions during the cold adaptation process and may play a general role in circadian phenomena associated with meristematic tissue. Phosphoinositide phospholipase C1 catalyses the majority of myo-inositol synthesis required for plant growth and development, acts as a repressor of programmed cell death and protects plant cells against cell death under high light intensity or long days. At3g14840-like protein belongs to the LRR-RK family, which may work in defense response or anther development (Zhao et al., 2002). Thus, reduced expression of these important genes in floral buds would affect microsporogenesis, which is very sensitive to various abiotic stress.

Moreover, accumulation of harmful wastes as in rice (Zhou et al., 2014) and up-regulation of key genes are also useful for searching for candidate genes. Three genes were up-regulated, including those encoding pollen-specific protein SF3, which has the potential to function as an actin filament bundling protein, glucanase (glucan endo-1,3-beta-glucosidase), and transcription factor WRKY family member, which is a frequently occurring elicitor-responsive cis-acting element. The other two up-regulated genes include ethylene-responsive transcription factor 1A-like, which acts as a transcriptional activator involved in the regulation of gene expression by stress factors, and pentatricopeptide repeat-containing protein (PPR) At1g07590, which has an unknown function. PPRs are involved in many post-transcriptional processes, such as splicing, editing, processing and translation within organelles, and some PPRs are well known as plant restorers-of-fertility (Delannoy et al., 2007).

### A working model for TGMS that affect tapetal differentiation and development

The genetic network for key genes that impact tapetal differentiation and development of *Arabidopsis* have now been characterized, and some are down-regulated in a certain male sterility (Ma, 2005; Aya et al., 2011; Zhu et al., 2011; Sharma and Nayyar, 2016). The earliest genes required for *Arabidopsis* cell division and differentiation into anther include *SPL, EMS1, TPD1*, and *ROXY2. Arabidopsis ems1* mutant anthers have excess microsporocytes, but no tapetal layer and male meiotic cytokinesis, resulting in the failure of tetrad microspores (Zhao et al., 2002). *AMS* and *DYT1* affect the later stages of development during tapetal maturation (Aya et al., 2011; Zhu et al., 2011; Sharma and Nayyar, 2016). At the final stage, *MS1* and *MYB80* are required for exine formation because *MS1* is involved in tapetal secretion and the exine structure and *MYB80* regulates *glucanase A6* transcription during callose dissolution (Vizcay-Barrena and Wilson, 2006; Zhang et al., 2007; Aya et al., 2011; Sharma and Nayyar, 2016). Many previous studies have suggested that down-regulation or knock-down of these genes leads to various male sterilities. However, both genetic regulation and cytological characterization of TGMS SP2S are different from these male sterilities. We found that expression of some tapetum-preferential genes was up-regulated, except for *BnSPL, BnTPD1*, and *BnROXY2*. Up-regulation of *BnEMS1* in SP2S may be caused by unknown TGMS genes that may affect tapetum formation, suggesting that TGMS genes may play an upstream regulatory role in normal tapetum development. Up-regulation of *BnMYB80, BnMS1*, and the tapetum marker gene *glucanase A6* was in line with the abnormal tapetal function of control callose dissolution and exine formation, as in the *Arabidopsis cdm1* (*CALLOSE DEFECTIVE MICROSPORE1*) mutant (Lu et al., 2014). It is possible that TGMS genes can regulate tapetum-necessary genes, such as *BnEMS1, BnAMS, BnDYT1, BnMYB80, BnMS1*, and *beta-glucanase A6*. Further experiments are needed to test whether these genes are direct targets of TGMS and/or other key proteins for tapetal development and function.

**Figure 9:**
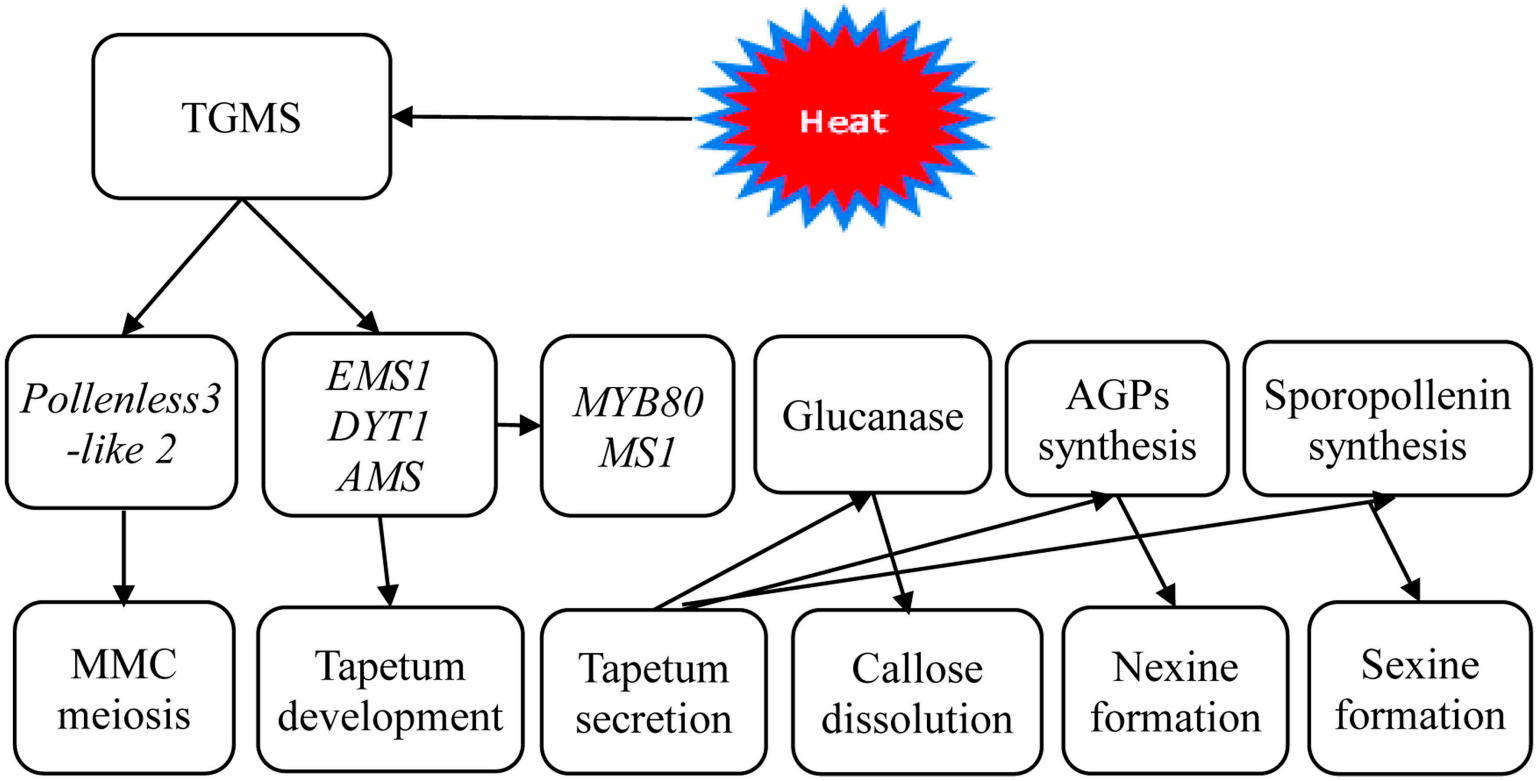
A scheme of genetic networks in TGMS SP2S. The functional network of TGMS in tapetum and microspore development in *B. napus* based on the analysis of differentially expressed transcripts and gene information from *Arabidopsis*.

Therefore, based on the results of these cytological and transcriptional analyses, a simple working model of TGMS male sterility in rapeseed was established (Figure 9). It is likely that the TGMS genes in SP2S affect key proteins that are required for microsporocyte meiosis and tapetum development. For example, BnEMS1, BnDYT1, and BnAMS resulted in defective tapetal cells during the microsporocyte stage. Up-regulation of *BnMYB80, BnMS1*, and *Glucanase A6* compensated for the delay of tetrad wall degradation and sexine formation by additional beta-glucanase and other related genes. However, the nexine was absent due to the shortage of AGPs.

## Conclusion

In summary, the results of cytological observation indicated that expression of TGMS in the SP2S line may be caused asynchronous meiosis of microsporocytes and premature breakdown of the fat tapetum. The aberrant transcriptional regulation in SP2S may disrupt the coordination of developmental and metabolic processes, resulting in defective tapetal cells and abnormal microspores. To our knowledge, this is the first investigation combining cytological and transcriptomic comparisons of temperature-induced male sterility in rapeseed. The ultrastructural abnormalities and altered gene expression in SP2S provided clues about TGMS genes for further study.

## Availability of supporting data

The data set supporting the results of this article is available in NCBI's Gene Expression Omnibus under accession GSE69638 and transcriptome shotgun assembly accession GDFQ00000000. Other supporting data are included in the article and its additional files.

## Author contributions

Conceived and designed the experiments, performed the bioinformatics analysis and wrote the paper: CY. Performed the experiments: XL, ZL, and JD. Contributed reagents/materials/analysis tools: JD, SH, and AX. All authors read and approved the final manuscript.

### Conflict of interest statement

The authors declare that the research was conducted in the absence of any commercial or financial relationships that could be construed as a potential conflict of interest. The reviewer IC and handling Editor declared their shared affiliation, and the handling Editor states that the process met the standards of a fair and objective review.

## Supplementary material

Figure S1 Flowers of near isogenic line SP2F and SP2S.

Figure S2 Heatmap of clustering of six samples and top 100 DETs. Heat maps showed top 100 DETs with log10 (RPKM+1) scale. Color intensities (from green to red shading) increased with elevated expression level, as indicated at upper left. F1, F2, F3, and S1, S2, S3 indicated different biological replicates of two groups of samples.

Table S1 Quality appraisal of digital gene expression tag profiling reads in the six samples.

Dataset S1 Differentially expressed transcripts (DETs) between SP2S and SP2F. A total of 465 DETs, including 303 up-regulated and 162 down-regulated in SP2S were listed in this dataset. DETs were selected with false discovery rate (FDR) ≤ 0.05 and fold change ≥2. Count number, RPKM value, fold change, gene sequence, GO terms, KEGG pathway, and Blast hits are shown.

Dataset S2 Result of GO term enrichment corresponding to differentially expressed transcripts of sample SP2S vs. SP2F. S gene is a significant gene by Fisher's exact test; TS gene is total significant genes; B gene is a gene in the pathway; TB gene is the total genes tested.

## Abbreviations

AGP: arabinogalactan protein
DET: differentially expressed transcript
DGE: digital gene expression
FPKM: fragments per kilobase of exon model per million mapped reads
GO: Gene Ontology
KEGG: Kyoto Encyclopedia of Genes and Genomes
MMC: microsporocyte
MS: male sterility
NCBI: The National Center for Biotechnology Information
NIL: near-isogenic line
RNA-seq: RNA sequencing
SNP: single nucleotide polymorphism
PGMS: photoperiod sensitive genic male sterility
TGMS: temperature or thermo-sensitive genic male sterility
TEM: transmission electron microscopy.
