# Supplementary figures and images for: TGMS in Rapeseed (Brassica napus) Resulted in Aberrant Transcriptional Regulation, Asynchronous Microsporocyte Meiosis, Defective Tapetum, and Fused Sexine
Source: Front Plant Sci. 2017 Jul 20;8:1268. doi: 10.3389/fpls.2017.01268 (PMC5517502; doi:10.3389/fpls.2017.01268)

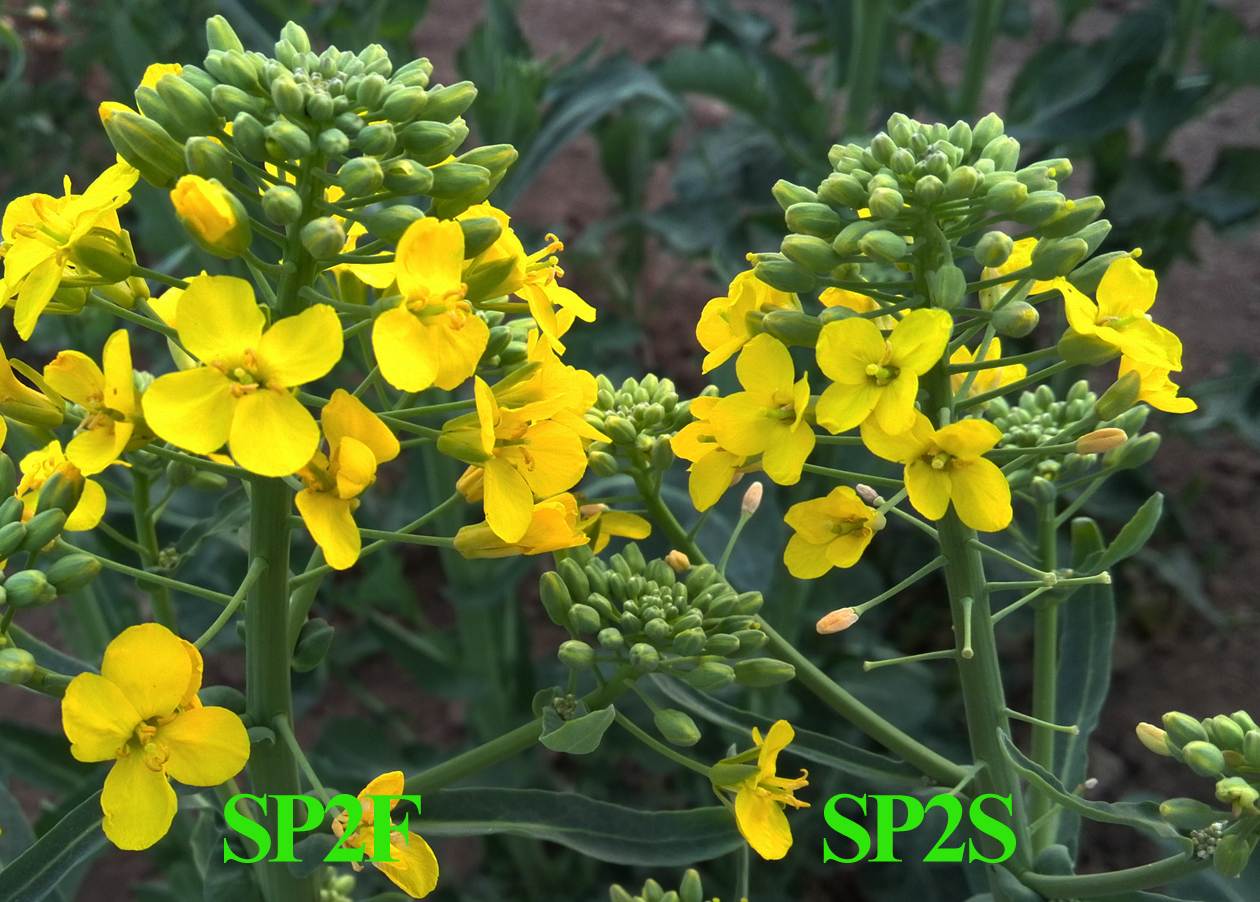

Supplement: Figure S1 — Flowers of near isogenic line SP2F and SP2S. [file Image1.JPEG]

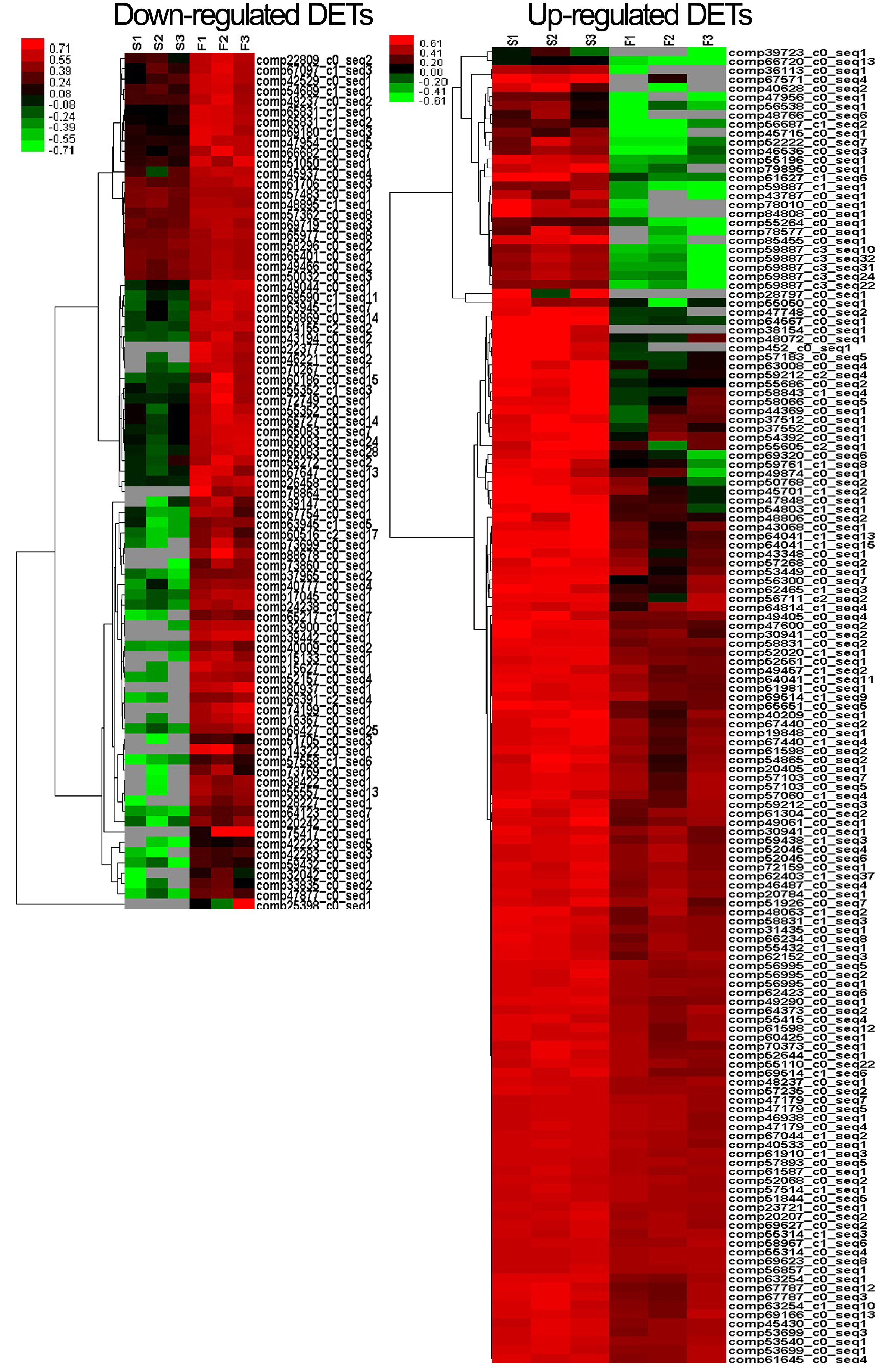

Supplement: Figure S2 — Heatmap of clustering of six samples and top 100 DETs. Heat maps showed top 100 DETs with log10 (RPKM+1) scale. Color intensities (from green to red shading) increased with elevated expression level, as indicated at upper left. F1, F2, F3, and S1, S2, S3 indicated different biological replicates of two groups of samples. [file Image2.JPEG]
