# Supplementary material for: TGMS in Rapeseed (Brassica napus) Resulted in Aberrant Transcriptional Regulation, Asynchronous Microsporocyte Meiosis, Defective Tapetum, and Fused Sexine
Source: Front Plant Sci. 2017 Jul 20;8:1268. doi: 10.3389/fpls.2017.01268 (PMC5517502; doi:10.3389/fpls.2017.01268)
Supplement: Table S1 — Quality appraisal of digital gene expression tag profiling reads in the six samples. [file Table1.DOCX]

**Supplemental Table S1 - Supplemental Table S1 - Quality appraisal of Digital Gene Expression tag profiling reads in the six samples**

| Sample | Raw read | Nucleotide base  (G) | Q20%* | Q30%* | GC% | Mapped gene | Max. RPKM | St.D of RPKM | Sum of RPKM |
| --- | --- | --- | --- | --- | --- | --- | --- | --- | --- |
| F1 | 12,045,328 | 0.43 | 98.4 | 94.4 | 47.0 | 111,268 | 10,660.4 | 51.2 | 994,823.4 |
| F2 | 7,241,994 | 0.26 | 98.4 | 94.2 | 47.6 | 106,915 | 6,800.8 | 43.5 | 994,784.2 |
| F3 | 8,298,346 | 0.30 | 98.6 | 94.8 | 48.0 | 106,274 | 7,856.5 | 43.0 | 960,476.7 |
| S1 | 8,594,447 | 0.31 | 98.4 | 94.2 | 47.5 | 109,003 | 13,686.0 | 59.7 | 1,000,456.5 |
| S2 | 9,762,561 | 0.35 | 98.5 | 94.7 | 46.4 | 111,160 | 10,539.9 | 56.9 | 1,031,773.8 |
| S3 | 8,643,180 | 0.31 | 98.4 | 94.2 | 47.2 | 108,391 | 12,997.9 | 58.1 | 996,543.5 |

* Q20 and Q30 were calculated by SolexaQA-BWA (http://solexaqa.sourceforge.net/)
